# Incipient Wear Detection of Welding Gun Secondary Circuit by Virtual Resistance Sensor Using Mahalanobis Distance

**DOI:** 10.3390/s23020894

**Published:** 2023-01-12

**Authors:** Daniel Ibáñez, Eduardo Garcia, Jesús Soret, Julio Martos

**Affiliations:** 1 Department of Electronic Engineering, Campus de Burjassot, Universidad de Valencia, 46100 Valencia, Spain; 2 Ford Spain, Poligono Industrial Ford S/N, 46440 Almussafes, Spain

**Keywords:** MFDC welding, wear monitoring, virtual sensor, Mahalanobis distance

## Abstract

Wear of the secondary of the welding gun, caused by mechanical fatigue or due to a bad parameterization of the welding points, causes an increase in quality problems such as non-existent welds or a reduced weld nugget size. In addition to quality problems, this defect causes production stoppages that affect the final cost of the manufactured part. Different studies have focused on evaluating the importance of different welding parameters, such as current, in the final quality of the welding nugget. However, few studies have focused on preventing weld command parameters from degrading or changing. This investigation seeks to determine the wear of the secondary circuit to avoid variability in the current supplied to the welding point caused by this defect and the increase in circuit resistance, especially in industrial environments. In this work, a virtual sensor is developed to estimate the resistance of the welding arm based on previous research, which has shown the possibility of detecting secondary wear by analysing the duty cycle of the power circuit. From the data of the virtual sensor, an anomaly detection method based on the Mahalanobis distance is developed. Finally, an integral system for detecting secondary wear of welding guns in real production lines is presented. This system establishes performance thresholds based on the analysis of the Mahalanobis distance distribution, allowing monitoring of the secondary circuit wear condition after each welding cycle. The results obtained show how the system can detect incipient wear in welding guns, regardless of which part of the secondary the wear occurs, improving decision-making and reducing quality problems.

## 1. Introduction 

Resistance spot welding (RSW) is widely used for joining metal sheets without extra material input. This is due it being possibly the fastest, cheapest, and most efficient joining method. These advantages make this method one of the most used technologies for joining metals in industries such as aerospace or automotive industries [1,2]. For example, currently, over 90% of assembly work in a car body is completed by RSW, a vital joining process for automotive production [3].

In the resistance spot welding process, two or more sheets of metal are pressed by means of the electrodes of the welding gun; this action can be carried out by a pneumatic cylinder or by a servomotor, depending on the type of welding. Once the necessary pressure is reached, the welding timer circulates the welding current through the contact metal sheets, generating the necessary energy to melt the metals. This energy generates following the basic Joule heat generation equation (Equation (1)):

(1) $$Q=I^{2}\cdot R\cdot t$$

Despite the simplicity of the heat generation equation, resistance spot welding is a complex process, as it involves different fields of study such as electromagnetism, electronics, thermodynamics, materials, and mechanics [4]. Therefore, due to the complexity of the process, there are many parameters that influence the final quality of the welding joint. The different parameters and influences on the final quality have been studied by different authors, highlighting the importance of pressure, current, electrode wear, welding shunt effect, or electrode misalignment [5,6,7,8]. Therefore, it is essential for the industry to guarantee the stability of the process to avoid the variations in the parameters that have an impact on welding. One of the defects in welding guns that causes production problems is the wear of the power circuit. Within this circuit, elements such as disparate screws, copper/aluminium arms of welding guns, diodes, and transformers are included.

### MFDC Welding

In resistance spot welding, there are two prevalent types of power sources responsible for controlling the way power is delivered to the load: three-phase medium-frequency DC power source and single-phase AC power source, both having different characteristics that generate a different effect in the way the nugget is formed [9]. First, in a single-phase AC power supply, two thyristors are connected in parallel, which allows current to pass during the positive semicycle through one of them, while in the negative semicycle, it passes through the other [10]. In this type of welding machine, the welding timer establishes the adjustment of the firing angle for each of the thyristors in each control cycle, starting from 0° to 180°. This firing angle control allows the welding timer to control the energy supplied to the welding point. The main characteristic of this type of welding is that in certain periods, the current is zero, which allows the welding nugget to cool, thus losing energy efficiency [11].

**Figure 1:**
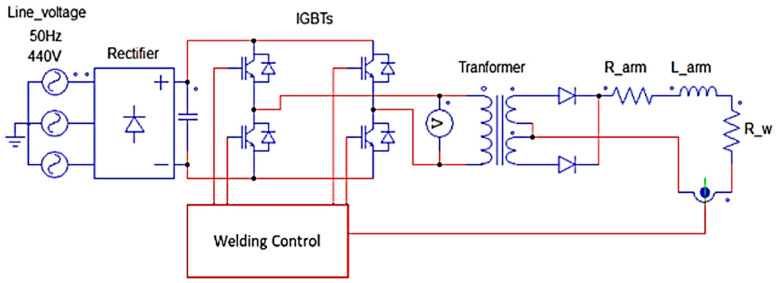
Schematics of MFDC Welder.

On the other hand, the medium-frequency three-phase DC power supply can be mentioned. This type of power supply is more complex, having four functional blocks: a three-phase AC power supply, a rectifier, an IGBT H-bridge inverter, and, finally, the welding transformer, as shown in Figure 1.

Figure 1 shows the three-phase network voltage rectified to an approximate DC single-phase voltage, Udc. This DC voltage is chopped at the H-bridge, in such a way that the primary voltage of the transformer depends on the state of the IGBTs. When IGBTs Q1 and Q4 are on, the voltage in the primary of the welding transformer is +Udc, while when Q2 and Q3 are in conduction, the voltage in the primary is -Udc. However, when the parallel IGBTs are on simultaneously, the output voltage will be equal to 0. The conduction states of the IGBTs are controlled by the welding timer. This control is based on an SMPS (phase-shifted full-bridge); therefore, to control the energy supplied to the welding point, the welding timer modifies the duty cycle of the SMPS [12,13].

Compared with the previous type of single-phase AC power supply, this type of welding power supply supplies a continuous current to the welding point, which allows heat to be supplied to the metal sheets during the entire process. The MDFC welder usually works at frequencies close to 1000 Hz, which allows greater control over the output current, adjusting it throughout the process based on the energy supplied. For these reasons, this type of power supply has a higher energy efficiency than the single-phase AC power supply. Due to this fact, this type of power supply is usually used in industries with a high production of welded joints, such as the automobile industry, as it favours lower energy consumption and greater control over the welded joints [14].

In both types of power supply, the electrical current of the secondary circuit depends on the primary voltage, either through the control of the IGBTs for the MFDC or through the phase shift of the thyristors firing in single-phase AC, and on the secondary resistance. Consequently, variations in the resistances of the secondary circuit cause variations in the current applied to the weld. It is, therefore, essential to guarantee the correct performance of the different elements that make up this power circuit.

**Figure 2:**
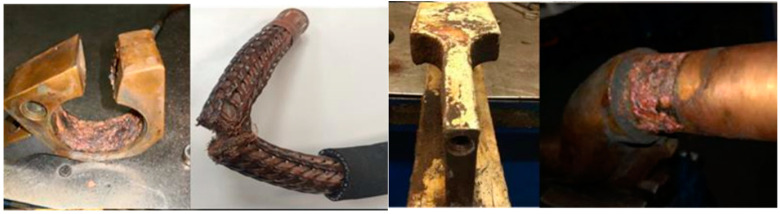
Typical wear in secondary circuit [15].

The wear that occurs in the power circuit, specifically that of the secondary circuit, has a very diverse origin and nature, as it occurs in different elements of the welding gun, as shown in Figure 2. The different wears that may occur are corrosion of the welding arms due to cooling water leaks, worn transformer pins, poor cooling or limescale, cracked arms caused by metal fatigue, clogged chilled braids, or thermal strip lamination breakage. All these types of wear cause both quality problems due to the instability of the process and production problems due to production stoppages caused by the need to repair the worn elements.

The detection of tool wear in different machinery and processes has been widely studied, as manufacturing industries tend to be increasingly automated, which makes it essential to detect events that may appear throughout the machinery process. These studies could be classified into two groups: those that use equipment additional to that of the production process and those that are based solely on the data acquired from the machine. As an example of the sensor-based tool wear monitoring, Gonçalves et al. [16] proposed a wear detection method in CNC machines by means of image analysis acquired during the production process. Similarly, Zhixiong et al. [17] showed a method for tool wear detection using audio signals together with machine learning, showing promising results for its real application in production lines. However, the main disadvantage of these methods is the need to purchase and install the additional equipment necessary to perform wear monitoring.

Second, due to the increasing availability of production data in the industry, new research is focused on sensorless wear monitoring. This type of monitoring usually requires greater knowledge of the process and a higher cost of data processing. For example, Zhang et al. [18] showed a complete analysis of the operation of the milling machine and, based on the monitoring of the physical model, showed a method of detecting wear and tear of the machine. In the same way and from the knowledge of the production lines, Garcia et al. [19] presented a method for the detection of wear of mechanical elements of the production lines by means of the analysis of the time used by the mechanical element to carry out the assigned task.

However, despite the growing demand for solutions for real-time detection of machinery wear in the industry, the different studies carried out in the field of resistance welding do not propose a real solution for the detection of wear in welding guns. As previously mentioned, many studies show the influence of the parameters on the final quality of welding. Similarly, many studies focus on the influence of mechanical problems and weld quality. In relation to the wear of the machine, the different studies have focused on the detection of the wear of the electrodes as these are a fundamental piece in the application of pressure and current to the welding point [20,21]. Nevertheless, at this time, there is no research that focuses its study on the detection of wear not only of the electrodes but of all the elements involved in the secondary circuit of the resistance welding gun.

Due to the absence of research on the determination of secondary wear in resistance welding guns, this gun variable cannot currently be monitored in real time. Consequently, for wear detection, welding guns are periodically inspected for signs of wear or damage. This can be performed visually, by looking for cracks, chips, or other signs of damage to the gun’s components. Additionally, the gun’s performance can be monitored over time to detect any changes that may indicate wear, such as a decrease in welding speed or accuracy, that is, once a consequence has already occurred in production. In some cases, it may be necessary to disassemble the gun and inspect its components more closely to detect wear.

Therefore, the main problem resides in the non-existence of an adequate method for the detection of wear in the secondary in the production lines, showing excessively high costs associated with its detection-repair. This leads to focusing the research on the search for a method capable of performing this monitoring of secondary wear automatically and efficiently.

## 2. Background and Objectives

In this section, studies carried out previously for the detection of secondary wear are presented first. These studies serve as a useful context and will serve as a starting point for this research. The objectives set out in our own research are presented below. These objectives are based on the research carried out previously and seek to solve the drawbacks of the previous solutions identified in these studies. In summary, this section addresses both the background of the research and the objectives that have been established in our study.

### 2.1. Previous Research

In previous investigations, a method for detecting problems in the secondary circuit of the welding guns was investigated by analysing the existing data in the welding timer (see [5]).

Starting from the existing data in the welding timer, it was verified that an increase in the variation in the wear conditions of the secondary had an influence on the control values of the welding timer. Specifically, the SMPS duty cycle history was analysed.

**Figure 3:**
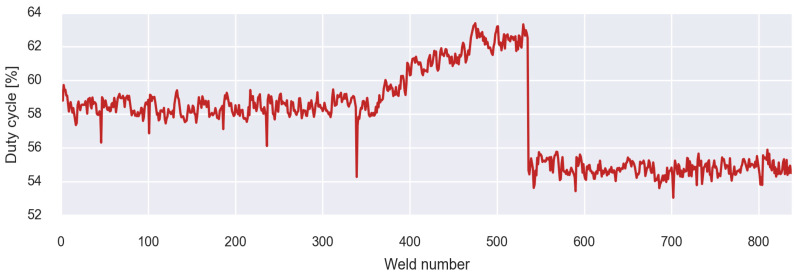
Analysis of secondary wear through duty cycle.The main problem with this method is that not all elements that wear cause a drastic increase in the overall resistance of the secondary, but sometimes, if the resistance of the arm is high, an increase in wear in a low-resistance element does not cause a noticeable increase in duty cycle, being difficult to detect it with the initial method.

Based on the duty cycle analysis, it was observed that as the welding arm wear increased, the welding timer corrected the IGBT control signal values. Figure 3 shows the evolution of the duty cycle in a pistol that presents secondary wear problems. In the first period from weld point 0 to the point 350 approximately, the duty cycle values are stable. From this moment on, the value of the duty cycle tends to increase up to the welding point 550 where the fault occurs. After carrying out the repair, the value of the duty cycle decreases and returns to behaving stable. Therefore, it was determined that by analysing these values, it is possible to detect wear in the welding arms. After establishing this relationship, an initial alarm detection system based solely on real-time monitoring of the SMPS duty cycle was established. This alarm system bases detection on the interquartile range rule [22].

Consequently, the above methods fail to fully respond to the existing problem, leaving out of detection those cases in which the resistance that wears out is much lower than the global resistance of the secondary. This makes it necessary to continue these lines of research to improve the previous methods in order to achieve a total detection of secondary wear.

### 2.2. Research Objectives

The main objective of this research is to improve the initial method for the early and effective detection of the wear of the secondary power circuit’s elements of the welding guns.

As mentioned, as the secondary resistance increases, the duty cycle increases to correct the current reduction in the welding tips, but sometimes, the resistance of the welding arm is much greater than the welding resistance. This means that when severe wear is generated within the elements that are part of the welding resistance, this will not cause a drastic increase in the total resistance of the secondary. As a consequence, the duty cycle will not increase considerably, thus making it difficult to detect wear using the alarm method described above.

As a result, the objective of this research is to improve the wear detection system based on the theoretical basis of the previous investigation, assuming that when wear exists, the resistance of the element in poor condition increases. To do this, and in accordance with the disadvantages of the previous system, the analysis will no longer start from the duty cycle but from the circuit’s own resistances, estimating them from the existing data in the welding timer, that is, from a virtual sensor of the resistances of the secondary.

## 3. Materials and Methods

### 3.1. Virtual Sensors for Resistance Estimation

A sensor can be defined as a device capable of measuring a physical magnitude and converting it into a signal that can be processed by the observer or instrument. Sensors can be divided into two categories: physical sensors and virtual sensors. Virtual sensors, also known as smart sensors or estimators, take readings from real physical sensors and calculate the outputs using some process models. This type of sensor is used due to the fact that they are flexible, cheap, and can perform measurements on elements that are difficult to measure due to their complexity [23].

**Figure 4:**
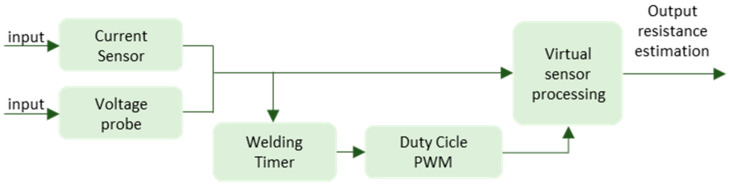
Conceptual summary of the virtual sensor for the estimation of the resistance of the secondary.

In this particular case, the estimation of the resistance of the secondary circuit of the welding gun is sought by means of the existing data in the welding control, such as the current, the voltage of the electrodes, and the duty cycle of the IGBTs, as can be seen in Figure 4.

The secondary circuit can be schematically reduced into two resistances, one corresponding to the resistance of the welding gun arm and the different elements that compose it, and another corresponding to the resistance of the electrode. The electrode resistance can be easily obtained from Ohm’s law, as the values of current and voltage drop are available in the welding timer. However, to obtain the resistance of the arm, only the value of the current that passes through the arm is available in the welding timer. Therefore, to obtain the value of the resistance of the welding gun arm, it is necessary to obtain first the other variables necessary for the calculation, specifically, the value of the voltage drop in the welding arm.

The secondary voltage can be calculated by decomposing the circuit in Figure 1 [24]. First, the rectifier converts the three-phase AC into a single-phase DC according to Equation (2):

(2) $$V_{rectifier}=\frac{1}{\frac{\pi }{3}}\int_{\frac{\pi }{3}}^{\frac{2\pi }{3}}V_{line}\sin\left(\omega t\right)d\left(\omega t\right)=\frac{3}{\pi }V_{line}$$

Then, starting from the behaviour equations of the SMPS and together with Equation (2), the secondary voltage can be expressed as a function of the duty cycle as:

(3) $$V_{sec}=2\left(\frac{N_{s}}{N_{p}}\right)V_{rectifier}\cdot D_{cycle}=\frac{\frac{6}{\pi }V_{line}D_{cycle}}{N}$$

Consequently, and starting from the variables obtained by the welding timer throughout the welding process, the value of the resistance of the welding arm can be estimated from Equation (4). This value of the resistance is estimated as it is assumed in this investigation that the power factors, the reactance, and the circuit losses that cause an error in the calculation of the resistance value remain stable within each particular welder. Therefore, it is considered unnecessary to calculate the exact value of the resistance.

(4) $$R_{arm}=\frac{V_{arm}}{I_{sec}}=\left(\frac{\frac{\frac{6}{\pi }V_{line}D_{cycle}}{N}-V_{weld}}{I_{sec}}\right)$$

**Figure 5:**
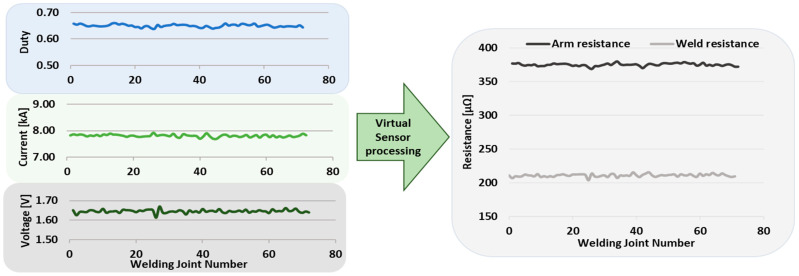
Virtual sensor operation. Input and output signals.

Summarizing, from the schematic of Figure 4 and Equation (4), Figure 5 of the operation of the virtual sensor is obtained. In Figure 5, it can be seen as a summary that the output signal of the system, that is, the resistance of the arm and the welding resistance, is calculated from the existing variables in the welding timer, current, welding voltage, and duty cycle.

### 3.2. Incipient Wear Detection

Once the variables of the virtual sensor on which the analysis is going to be carried out have been obtained, a method for the detection of incipient wear for resistance welding guns is proposed.

In this case, it is intended to detect anomalies in the resistance data to detect incipient wear of the secondary circuit. An anomaly is defined as data that deviate from the normal behaviour of a series of data and, in this research, it is decided to use the analysis of the Mahalanobis distance for the detection of anomalies and, therefore, for the detection of wear in the secondary circuit.

The Mahalanobis distance (MD) is the measure of the effective distance between a point and a distribution [25], being effective in multivariate data. These result due to the use of the covariance matrix of variables to find the distance between data points and the centre, according to Equation (5). That is, Mahalanobis calculates the distance between point “P_1_” and point “P_2_” considering the standard deviation. MD, therefore, provides good results with outliers being considered multivariate. To find these outliers by MD, the distance between each point and the centre in n-dimensional data is calculated and outliers are found considering these distances. MD detects outliers based on the distribution pattern of data points [26,27].

(5) $$D^{2}={\left(X_{p}{}_{1}-X_{p}{}_{2}\right)}^{T}\cdot C^{-1}\cdot \left(X_{p}{}_{1}-X_{p}{}_{2}\right)$$

where *C* is the covariance matrix and *X_pn_* represents coordinates of observations in n-dimensional space.

(6) $$d\left(p,q\right)=\sqrt{{\left(p_{1}-q_{1}\right)}^{2}+{\left(p_{2}-q_{2}\right)}^{2}+..+{\left(p_{n}-q_{n}\right)}^{2}}$$

Euclidean distance, *d*, Equation (6), is also commonly used to find the distance between two points (*p* and *q*) in two- or higher-dimensional spaces [28]. However, MD uses the covariance matrix unlike Euclidean. This means that MD can be used with correlated variables even if the points do not have the same scale.

**Figure 6:**
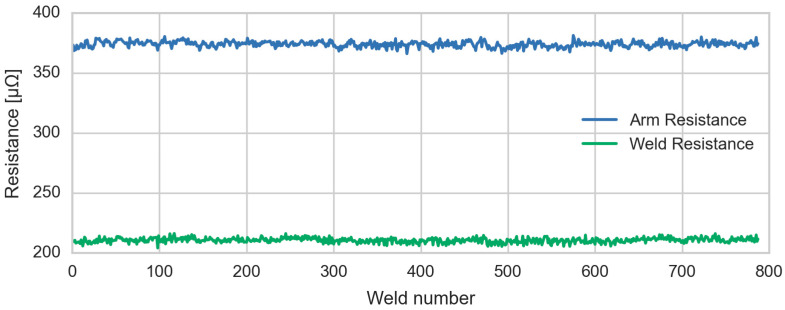
Virtual Sensor Resistance Output Values.

Figure 6 shows the resistance data of the arm and the welding obtained from the virtual sensor for a welding gun of a production line. These data are used to calculate both the Mahalanobis distance and the Euclidean distance. Once the distances have been calculated, pre-alarm and alarm thresholds are established. These anomaly thresholds are established bearing in mind the distribution of the calculated distance according to Equation (7).

(7) $$Th_{pre-alarm}=Q3+1.5\cdot IDR\;\;\;\;\;\;\;\;\;\;\;Th_{alarm}=Q3+3\cdot IDR\;$$

where *Q*3 represents the value of the third quartile and *IDR* the difference between the first decile and the ninth decile.

**Figure 7:**
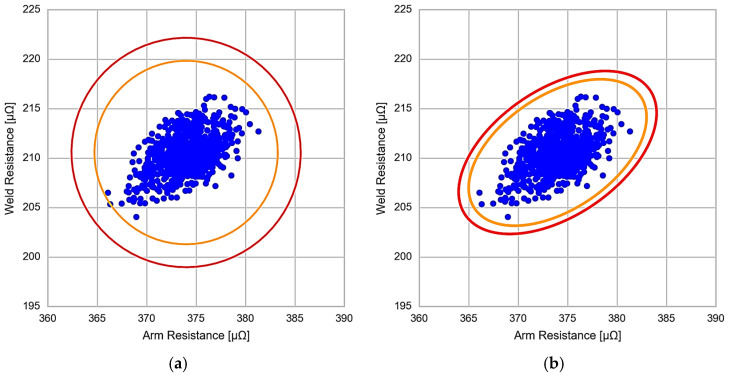
Threshold comparison. (**a**) Euclidean distance. (**b**) Mahalanobis distance.

Figure 7 shows the calculated thresholds using the Euclidean distance (a) and the Mahalanobis distance (b). Comparing both results, in the case of the Euclidean distance, the thresholds are set in a circle, causing a poor fit with the data dispersion. However, using the Mahalanobis distance, and therefore, the correlation between both variables, the thresholds are set elliptically, which allows further adjustment of the thresholds to the correlated behaviour of the data [29].

Considering the result obtained from the thresholds, it is considered that for this study in which there is a correlation or linearity between both variables, the most appropriate method for detecting anomalies is MD. However, to validate this statement, the same welding gun in which an anomaly begins to appear is evaluated.

**Figure 8:**
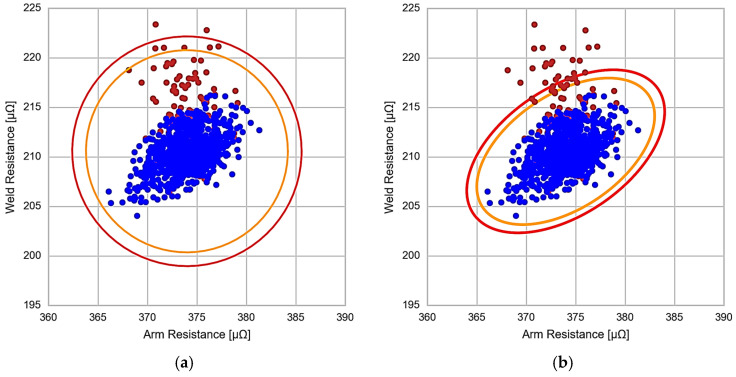
Incipient wear detection. (**a**) Euclidean distance. (**b**) Mahalanobis distance.

Figure 8 shows the previous data (blue dots) together with the new data corresponding to the appearance of wear in some mechanical element of the secondary circuit (brown dots).

Analysing the data obtained in Figure 8, some conclusions can be drawn. First, in Figure 8a, which corresponds to the detection of anomalies by means of the Euclidean distance, despite the existence of anomalies, many of the data corresponding to the anomaly, brown points, are within the thresholds alarm and pre-alarm. However, a small number of points are above the thresholds. Therefore, this method is valid to detect anomalies but not in an incipient way, as it is not capable of detecting the first initial values of the anomaly.

**Figure 9:**
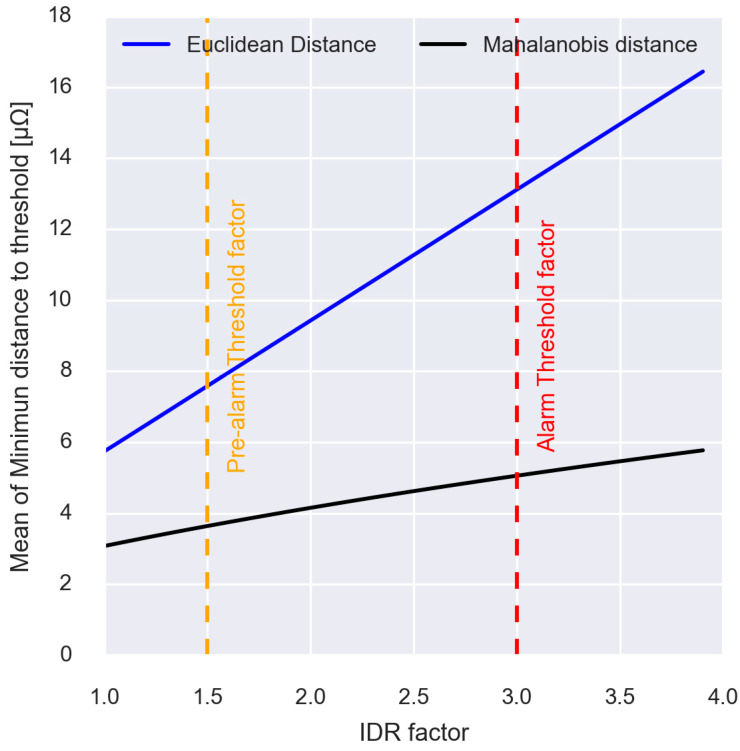
Comparison of the average minimum distance between the points and the closest threshold for the Euclidean distance and the Mahalanobis distance.

On the other hand, Figure 8b shows the anomaly detection results using the Mahalanobis distance. From the results, it can be seen that many points are located outside the alarm and pre-alarm thresholds compared to the previous case. This behaviour suggests that the Mahalanobis distance method would be better for early anomaly detection. To conclude with the analysis of both methods, Figure 9 represents the average of minimum distances from each point with its closest alarm threshold for each of the methods and as a function of the multiplying factor of the inter-decile range of Equation (7). This value is used to determine which of the methods finds the anomaly earlier, or what is the same as the incipient wear of the secondary circuit.

Several conclusions can be drawn from Figure 9. Throughout the different IDR multiplication factors that can be set for the calculation of the thresholds, the average minimum distance to the nearest threshold is lower by the Mahalanobis distance, which, therefore, results in a better fit of the thresholds to the existing data population. In the same way, it is observed that if an increase in the IDR multiplying factor is necessary to that established in Equation (7) to avoid false positives, in the case of the Euclidean distance, the values of the average minimum distance increase almost linearly with the increase in the factor, which increases the difficulty of early detection of the abnormality. Consequently, it is established that the most adequate method for the incipient detection of secondary wear is the one based on the Mahalanobis distance between the resistance of the welding arm and the welding resistance.

### 3.3. Wear Detection System

Once the method used for the detection of incipient wear has been established, a system is designed to apply the method autonomously not only to a welding gun but also to an indeterminate number of them. The detection system is mainly divided into three stages: calibration and obtaining of initial threshold, analysis of new incoming data, and recalibration.

**Figure 10:**
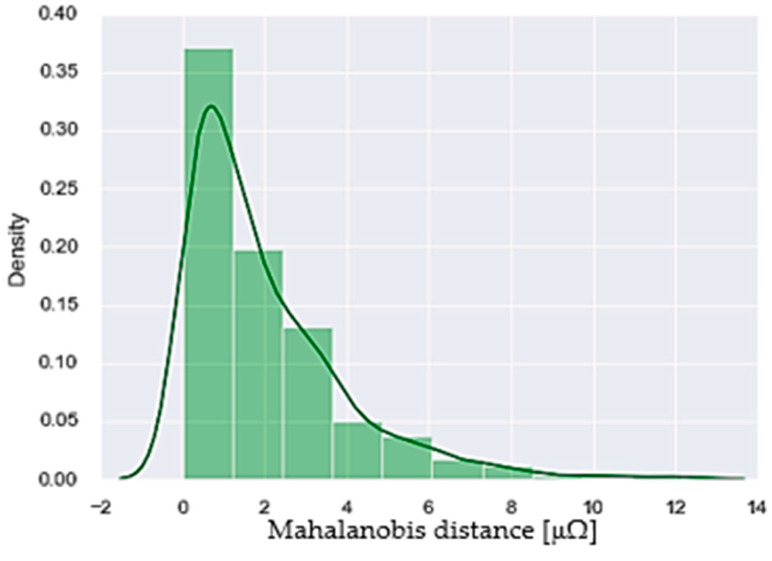
Mahalanobis distance distribution.

Then, Algorithm 1 is followed for calibration. Current, voltage, and duty cycle values are obtained to calculate the arm resistance and welding resistance. Once the resistance values are obtained, the Mahalanobis distance is calculated, and the alarm and pre-alarm thresholds are established by the Mahalanobis distance distribution (Figure 10) and according to Equation (7). These alarm and pre-alarm values are stored to be used in the analysis stage.
**Algorithm 1:** Threshold Calibration.**Input:** V_weld_, I_sec_ and D_cycle_ **Output:** Al (Alarm Thershold), Pr (Prealarm Threshold), C   1.Calculate R_arm_ and R_weld_ resistance according to Equation (4).  2.Calculation of the inverse covariance matrix of R_arm_ and R_weld_.
$\;\;\;\;C=\left[\begin{array}{cc} Var\left(R_{arm}\right) & Cov\left(R_{arm},R_{weld}\right)\\ Cov\left(R_{weld},R_{arm}\right) & Var\left(R_{weld}\right) \end{array}\right]$
  3.Calculation of the Mahalanobis distance according to Equation (5).  4.Determination of the alarm and pre-alarm thresholds based on the Mahalanobis distance distribution.


After establishing the pre-alarm and alarm values for each welding gun, Algorithm 2 is carried out for the analysis of anomalies for the new incoming data collected from the production line. This algorithm calculates the resistance values again and the Mahalanobis distance between both variables starting from the covariance matrix calculated by Algorithm 1 for each welding gun. Once the distance has been calculated, each of the welding points is evaluated with the alarm and pre-alarm thresholds.
**Algorithm 2:** Analysis of new incoming data.**Input:** V_weldnew_, I_secmew_, D_cyclenew_, Al, Pr and C **Output:** Alarm/Prealarm warning   1.Calculate R_arm_ and R_weld_ resistance according to Equation (4).  2.Calculation of the Mahalanobis distance (D) according to Equation (5) using the old covariance matrix (C).  3.Determination of alarm/pre-alarm status:
    If D > Al then “Alarm”    If D > Pr and D < Al then “Pre-alarm”

Finally, to improve the detection method, a recalibration process is carried out after correcting the mechanical failure that caused the alarm of a certain welding gun. In addition, Algorithm 3 is exescuted periodically so that if the new data present thresholds lower than those previously calculated, they are updated. This makes it possible to better adjust the thresholds and detect wear earlier.
**Algorithm 3:** Recalibration after alarm.**Input:** V_weld_after_alarm_, I_sec_after_alarm_, D_cycle_after_alarm_, Al, Pr and C **Output:** Al, Wn, C   1.Calculate R_arm_ and R_weld_ resistance according to Equation (4).  2.Calculation of the Mahalanobis distance (D) according to Equation (5) using the new covariance matrix (C).  3.Determination of the alarm and pre-alarm thresholds based on the Mahalanobis distance distribution,  4.Comparation between new and old threshold:
    If Al_new_ < Al and Pr_new_ < Wn then:       Al = Al_new_
       Pr = Pr_new_

**Figure 11:**
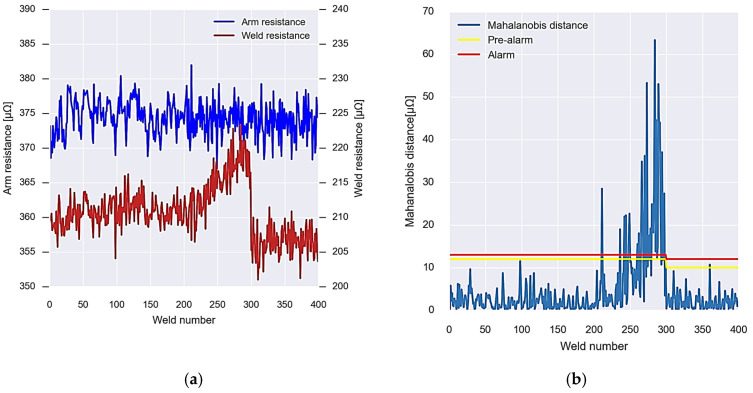
Graphic summary of the operation of the wear detection system algorithms. (**a**) Resistances obtained from the virtual sensor. (**b**) Calculated Mahalanobis distance and alarm and pre-alarm thresholds.

As a summary, Figure 11 shows the operation of the algorithms mentioned above. Figure 11a shows the resistance values of the arm and the weld, while Figure 11b shows the values of the Mahalanobis distance between both variables. In the first place, the initial data are used to calculate the covariance matrix and the alarm thresholds (Algorithm 1). The data show a normal behaviour, without detecting any anomaly until approximately weld number 200. From this moment on, the data begin to experience a growing anomaly. This anomaly is detected by the system using Algorithm 2. After executing the appropriate maintenance operations, approximately at weld number 300, the alarm system is recalibrated with the new data, calculating the new covariance matrix and the new detection thresholds (Algorithm 3).

## 4. Results

The results section shows the results of the application of the proposed system in a real factory. This section presents the block diagram of the data collection system within a real factory, together with three success stories in which the virtual sensor and the proposed algorithms have been used to detect different types of anomalies in the processes of the factory. Each of these cases demonstrates the effectiveness of the system in detecting anomalies and its ability to be used in real production lines.

**Figure 12:**
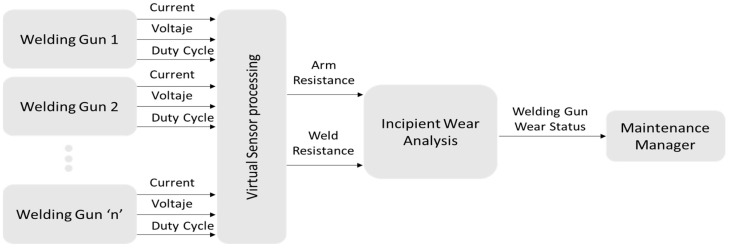
Flow diagram of the operation of the incipient wear detection system in welding guns.

This system is designed to be implemented in the manufacturing industry, specifically, its operation is tested in an automotive production factory. The data collection system is presented in the block diagram of Figure 12. Each of the welding guns collects current, welding voltage, and duty cycle for each weld joint made, and these data are sent to the base welding data.

The data from the welding database goes through the virtual sensor processing in order to obtain the resistance values. Once the resistances are calculated, they are stored in the analysis database. To reduce the number of data stored in the database, the average resistance data between milling cycles are analysed.

The data from the analysis database are used to calculate the alarm and pre-alarm thresholds, as shown in the previous section. Finally, once the thresholds have been established, the new data are analysed, the wear status of the secondary of each of the welding guns is labelled, and an alert message is sent to the operator in charge of each of the analysed equipment.

In total, this system has been tested on six hundred and fifty different welding guns. The welding guns analysed are of the MFDC type, differing in two types of pressure application, either by a pneumatic cylinder or by a servomotor.

The results shown below are extracted from the six hundred and fifty welding guns analysed; specifically, three real cases of welding guns installed in production lines that have detected wear are presented. These three results show the total number of patterns that can occur in welding guns: increase in welding arm resistance, increase in welding resistance, and increase in both resistances.

### 4.1. Gun 1: Welding Arm Wear

**Figure 13:**
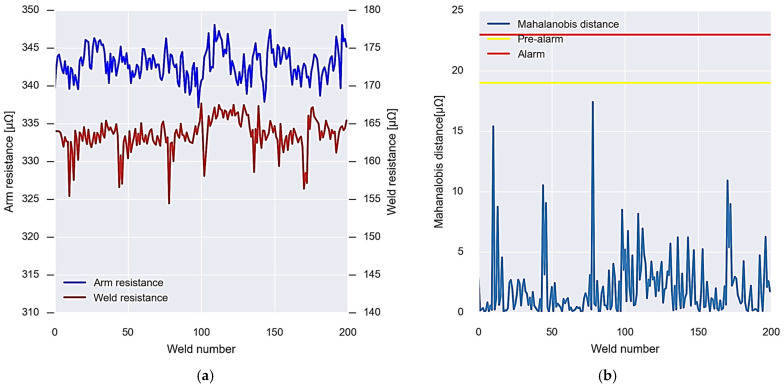
Operation of the initial calibration algorithm for gun 1. (**a**) Data of the resistances used for the calibration. (**b**) Calibration result, Mahalanobis distance, and alarm thresholds.

In this section, the results of the calibration of welding gun 1 are presented following the first calibration algorithm shown in the previous section. Figure 13 shows the resistance data of the arm and the welding of the gun used for calibration; in total, 200 welding points made by that welding gun are used. In this way, the dynamic behaviour of the process is considered, as well as the own variations due to the wear of the capsules and the change of the same. The resistance of the arm has an average value of 340 μΩ, while the resistance of the weld has an average value of 160 μΩ.

Finally, Figure 13 shows the thresholds obtained by means of the calibration algorithm as a function of the Mahalanobis distance between the two resistors. If the data are within the thresholds, the calibration is considered to have been successful. Otherwise, a new calibration must be performed.

**Figure 14:**
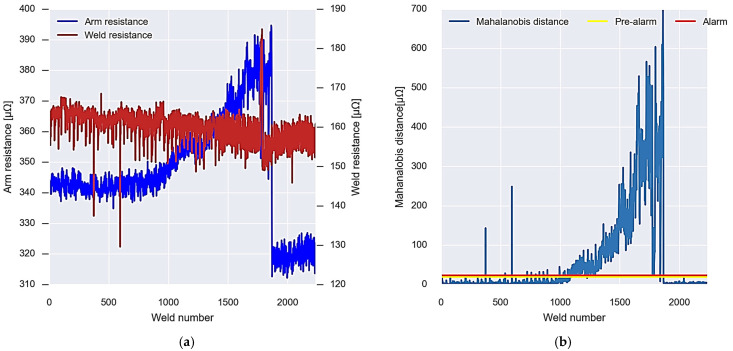
Gun 1 alarm system operation. (**a**) New incoming resistance data. (**b**) Analysis and recalibration of alarms.

Once the alarm and pre-alarm thresholds for gun 1 have been set, the new data coming from the welding gun virtual sensor are analysed in real time. Figure 14a shows the values of each of these arm and weld resistance data.

For these data, the Mahalanobis distance is calculated considering the covariance matrix obtained in the calibration. Once the Mahalanobis distance is calculated, it is evaluated together with the set thresholds, and it is figured out whether it is an alarm or not. In this case, an alarm is experienced up to weld point 1800, where the values drop, and a recalibration is performed using Algorithm 3.

### 4.2. Gun 2: Welding Resistance Wear

**Figure 15:**
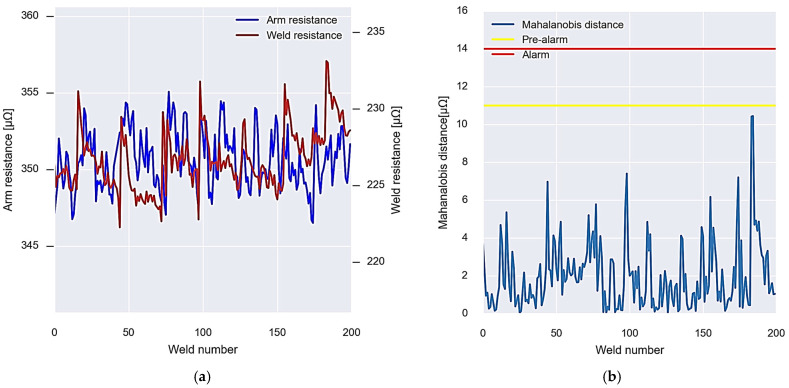
Operation of the first calibration algorithm for gun 2. (**a**) Data of the resistances used for the calibration. (**b**) Calibration result, Mahalanobis distance, and alarm thresholds.

As in the case of gun one, for gun two, a first calibration is carried out with 200 values. Figure 15a shows the resistance values of both the arm and the welding of the gun. The arm resistance has an average value of 350 μΩ, while the welding resistance is 230 μΩ.

As in the previous case, the Mahalanobis distance between the arm and weld resistance data is calculated and evaluated together with the alarm and pre-alarm thresholds set during calibration. In this case, no point is above the established thresholds, writing down that the calibration has been successful and that the gun is in good condition (Figure 15b).

**Figure 16:**
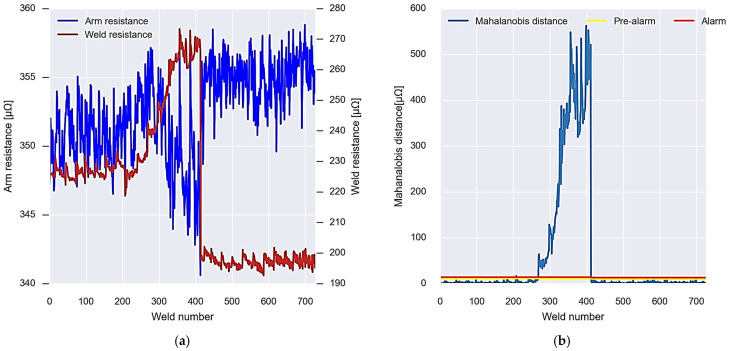
Gun 2 alarm system operation. (**a**) New incoming resistance data. (**b**) Analysis and re-calibration of alarms.

In Figure 16a, the data can be seen in real time after the first calibration of welding gun 2. In this case, it can be seen how the resistance data have greater variability, but despite this, the Mahalanobis distance remains below the alarm thresholds up to the point where the alarm occurs (Figure 16b). In the same way as in the previous case, after the alarm period has elapsed, the recalibration of the algorithm is carried out.

### 4.3. Gun 3: Welding Resistance and Arm Resistance Wear

**Figure 17:**
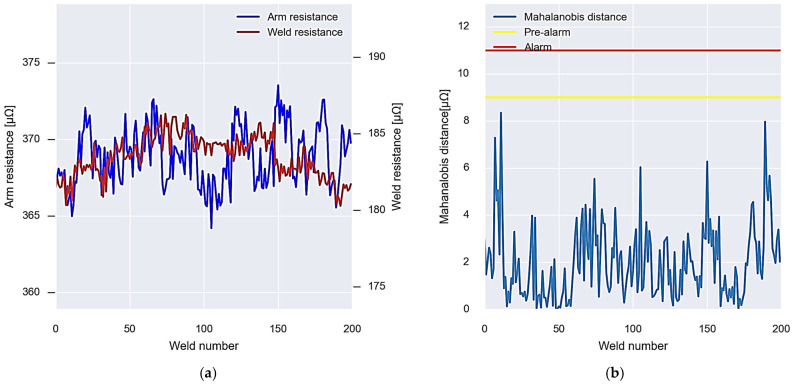
Operation of the first calibration algorithm for gun 3. (**a**) Data of the resistances used for the calibration. (**b**) Calibration result, Mahalanobis distance, and alarm thresholds.

Lastly, for gun 3, the same calibration is carried out as in the two previous cases, taking the initial 200 values of resistance of the arm and welding (Figure 17a). These values allow you to calculate your covariance matrix and the alarm and pre-alarm thresholds (Figure 17b).

**Figure 18:**
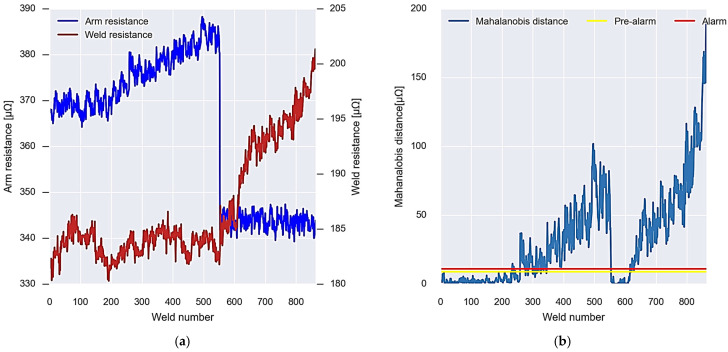
Gun 3 alarm system operation. (**a**) New incoming resistance data. (**b**) Analysis and recalibration of alarms.

In the same way, this welding gun presents an evolution in which the Mahalanobis distance data between the resistors exceed the thresholds defined by the calibration. Figure 18a shows the evolution of the resistance values of the arm and welding resistance after carrying out the initial calibration. In this case, around weld point 250, it is first exceeded; once the fault is repaired, the Mahalanobis distance is recalibrated to return to values above the alarm thresholds; after weld point 100, the alarm threshold is exceeded again, without observing any type of repair (Figure 18b).

## 5. Analysis and Discussion of Results

The data obtained by the virtual sensor for gun 1 show that after having performed the calibration based on the Mahalanobis distance and setting the behaviour thresholds, an alarm is produced around point 1000 as the data exceed the established thresholds, both alarm and pre-alarm. The distance between the arm strength data and the weld continues to increase until the defect repair is performed around weld point 1800 (Figure 14).

After the repair, Algorithm 3 is carried out, which recalculates the covariance matrix and the new alarm and pre-alarm thresholds. Observing the evolution of the resistance of the arm and the resistance of the welding in the figure, it can be determined that this alarm is caused by the resistance of the welding arm and, therefore, wear will occur in the copper strips, in the connections of the transformer, or in the connecting bolts of the arms.

**Figure 19:**
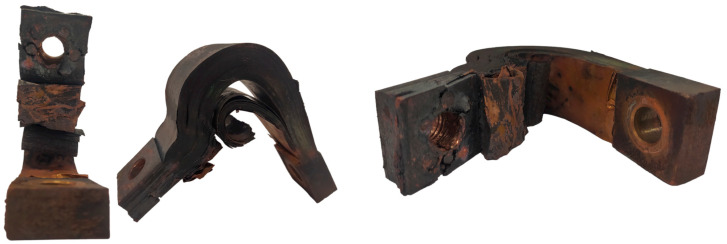
Real case of detection-worn copper strip. Cut sheets.

After the system sent the alarm and in order to repair the existing defect, the operators carried out a visual inspection of the welding gun to carry out the repair and verified that the copper strip was in poor condition. The strapping pins were burned, and the sheets had started to break, causing an exponential increase in resistance (Figure 19). This explains the increase in the Mahalanobis distance seen in the data and the generation of the alarm.

If the data obtained for gun 2 is observed (Figure 16), it can be seen that an alarm occurs at weld point 250. This is because the Mahalanobis distance data exceed the established thresholds. This situation is maintained until weld point 400, where a repair of the defect is performed. Analysing Figure 16, it can be seen that the increase in Mahalanobis distance is related to the increase in weld resistance. This suggests that the worn items are the electrodes, electrode holders, or hardware in this section of the welding gun. Therefore, repair should focus on these components to correct wear and reduce the Mahalanobis distance. After the repair of the worn element, a recalibration of the welding gun wear detection algorithm is performed to adjust and reduce the Mahalanobis distance. As a result, the data return to values below the alarm and pre-alarm thresholds.

Finally, the case of gun 3 (Figure 17) shows two different types of alarm. In the first place, it is observed that from weld point 250 to 550, the Mahalanobis distance data are above the alarm and pre-alarm thresholds due to the increase in the resistance of the arms. After this, a recalibration of the wear detection algorithm is performed and the data return to normal. However, around weld point 650, the Mahalanobis distance again exceeds the thresholds, but this time due to weld resistance. In this case, it is seen that the data continue to continuously increase without any repair or recalibration of the algorithm being performed.

In summary, the three cases analysed show the effectiveness of the welding gun wear detection system in real time. In the case of gun 1, the alarm occurs due to the increase in the resistance of the arms, which indicates that the wear occurs in the arms, the copper straps, the screws, the transformer terminals, etc. In the case of gun 2, an alarm occurs due to increased welding resistance, indicating that wear is occurring on the electrodes, electrode holders, or hardware in this section of the welding gun. In the case of gun 3, two different alarms are produced due to different causes of wear, both on the arm and on the welding resistance. In short, these cases prove the system’s ability to detect incipient wear in welding guns and to send alarms to operators so that they can intervene promptly and prevent welding gun failures and weld quality problems.

## 6. Conclusions

This article presents a system to detect the incipient wear of welding guns in real time. The main objective of this system is to improve efficiency and quality in the welding process by detecting early wear on the welding guns and allowing operators to intervene in a timely manner.

To achieve this goal, the system uses a virtual sensor that converts current, voltage, and duty cycle signals into arm resistance and weld resistance values. Then, the Mahalanobis distance between both resistors is calculated and, based on those values, sends alarms to the operators.

The system was tested in a real study in a car manufacturing factory in which 650 welding guns were tested. The results showed that the system is capable of accurately and opportunely detecting incipient wear both in the arms of the gun and in the part in charge of welding (electrodes, electrode holders, etc.).

Furthermore, the system has several advantages compared to other wear detection methods. In the first place, it is not based on visual inspections or on measurements carried out by operators, so it is not necessary to have personnel in charge of carrying out these actions. In addition, it is based on accessible data, which makes it easier to implement in factories. Secondly, it makes it possible to detect wear in real time, which makes it possible to intervene before failures occur in the welding gun, unlike current systems based on periodic maintenance.

The main disadvantage present in the proposed method is the need to have to recalculate the covariance matrices for each welding gun for both Algorithm 1 and Algorithm 3. This increases the computational cost and increases the probability of system execution failure due to, for example, the covariance matrix not being reversible. In this research, a comparison has been made between two different methods for calculating the distances between the resistors; however, it would be necessary to make a comparison with other, different methods to establish to a greater extent which ones present a more adequate behaviour for the detection of the secondary wear.

In short, this innovative system is an efficient and accurate way to detect incipient wear on welding guns. It can improve efficiency and quality in the welding process and has several advantages compared to other wear detection methods.

## Data Availability

Not applicable.
